# Endovascular treatments of acute pulmonary embolism in the post-fibrinolytic era: an up-to-date review

**DOI:** 10.1186/s13244-024-01694-9

**Published:** 2024-05-20

**Authors:** Nicolò Ubaldi, Miltiadis Krokidis, Michele Rossi, Gianluigi Orgera

**Affiliations:** 1 Department of Radiology, Sant’Andrea University Hospital La Sapienza, School of Medicine and Psychology, “Sapienza” - University of Rome, Rome, Italy; 2 1st Department of Radiology, School of Medicine, National and Kapodistrian University of Athens, Areteion Hospital, Athens, Greece

**Keywords:** Pulmonary embolism, Pulmonary thrombectomy, Aspiration device, Endovascular

## Abstract

**Abstract:**

Pulmonary embolism (PE) is a significant contributor to global cardiovascular-related mortality that mainly depends on the severity of the event. The treatment approach for intermediate and high-risk PE remains a topic of debate due to the fine balance between hemodynamic deterioration and bleeding risk. The initial treatment choice for intermediate-risk PE with hemodynamic deterioration and high-risk PE is historically systemic thrombolysis, but this approach is not always effective and carries a notable risk of severe bleeding. For such patients, various interventional treatments have been introduced to clinical practice, including catheter-directed lysis (CDL), ultrasound-assisted CDL, pharmacomechanical CDL, and aspiration thrombectomy. However, the optimal treatment approach remains uncertain. Encouraging outcomes have been presented assessing the novel endovascular treatments, in terms of reducing right ventricular dysfunction and improving hemodynamic stability, opening the possibility of using these devices to prevent hemodynamic instability in less severe cases. However, ongoing randomized trials that assess the efficacy and the association with mortality, especially for aspiration devices, have not yet published their final results. This article aims to offer a comprehensive update of the available catheter-directed therapies for PE, with a focus on novel mechanical thrombectomy techniques, assessing their safety and efficacy, after comparison to the conventional treatment.

**Critical relevance statement:**

This is a comprehensive review of the indications of use, techniques, and clinical outcomes of the most novel endovascular devices for the treatment of pulmonary embolism.

**Key Points:**

- Mechanical thrombectomy is an effective tool for patients with PE.
- Aspiration devices prevent hemodynamic deterioration.
- Catheter directed therapy reduces bleeding complications.

**Graphical Abstract:**

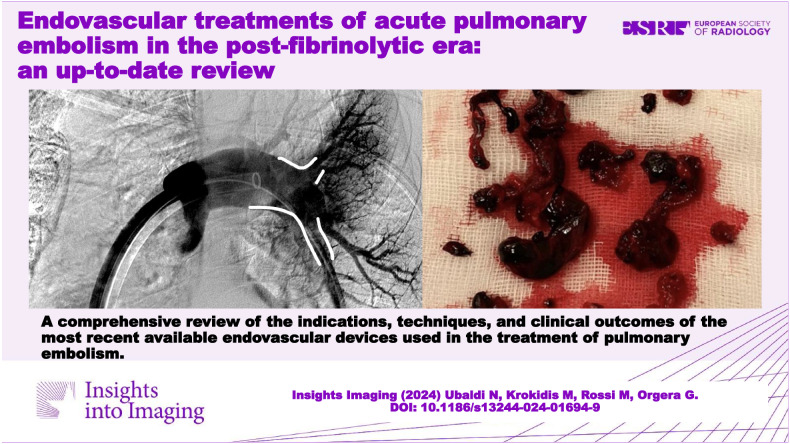

## Introduction

Pulmonary embolism (PE) is the third most common cause of cardiovascular-related death and is a significant contributor to global mortality [1]. Risk stratification of early mortality is necessary for the correct therapeutic management. PE is classified in: High risk (massive) when associated with hemodynamic instability, defined as the presence of either cardiac arrest, obstructive shock or persistent hypotension; Intermediate risk (segmental) pulmonary embolism severity index III-IV, right ventricular (RV) strain and elevated cardiac troponin levels, a condition which may evolve to hemodynamic deterioration; Low risk (subsegmental) when none are present [2]. In-hospital mortality for intermediate PE is approximately 2.9%, whereas 30-day mortality reaches 15% [3–8]. It was pointed out that particularly for intermediate risk PE there is a mortality rate that persists after discharge most likely because the patients receive only anticoagulation and in most cases no form of thrombolysis or other more advanced catheter directed or surgical treatment [7]. This underlines the fact that intermediate risk PE is underestimated, and more drastic initial measures are required for patient management. Indirect computed tomography venography with appropriate opacification of the pulmonary artery is the most widely used imaging method for the assessment of the extension of PE and for planning the most adequate treatment. In cases of hemodynamically unstable patients with high clinical suspicion of PE, direct catheter angiography may be directly performed for imaging and potential immediate treatment [9].

According to the European Society of Cardiology (ESC) 2019 guidelines, in patients with intermediate risk PE, anticoagulation should be initiated whereas systemic thrombolysis and catheter directed therapy (CDT) is reserved for those deteriorating to hemodynamic instability [2]. Under the term catheter directed therapy both catheter directed thrombolysis and catheter directed aspiration thrombectomy are included. Moreover, in patients with high-risk PE, systemic thrombolysis and anticoagulation should be immediately administered [2]. When systemic thrombolysis is contraindicated or has failed, catheter directed treatment may be offered (Table 1).

**Table 1:** Classification for the management of acute pulmonary embolism according to the 2019 ESC guidelines

Early mortality risk	Treatment recommendations (Class; Level)
Intermediate risk	Anti-coagulation (I A). Systemic thrombolytic therapy (I B) in case of hemodynamic deterioration. Percutaneous catheter treatment (IIa C) as an alternative to rescue thrombolytic therapy and in case of hemodynamic deterioration.
High risk	Anti-coagulation with unfractionated heparin (I C). Systemic thrombolytic therapy (I B). Percutaneous catheter treatment (IIa C) in case of failure or contraindication to thrombolytic therapy.

The overall catheter directed therapy procedure success rate, defined as obtaining hemodynamic stabilization and overall survival at hospital discharge, ranges at 87% [10] (Tables 2, 3).

**Table 2:** Summary of the benefits and pitfalls of each mechanical aspiration device

	INARI	ANGIOVAC	INDIGO
Advantages	Nitinol discs help trap the parietal thrombi; Innovative design allows improved manoevrability in tortuous vessels; Large caliber allows efficient thrombi removal also of bigger dimensions.	Reduced blood loss; Reaches other venous vessel districts and remove blood clots; Large caliber allows efficient thrombi removal.	Innovative design improved manoevrability in tortuous vessels; Reduced blood loss; Reduced vascular access complications or damage to the cardiocirculatory organs; Fastest mean time of utilization; Dual mechanism based on pressure and flow.
Disadvantages	Blood loss (New FlowSaver system needs to be validated); Damage to cardiovascular structures due to the large caliber.	Lack of evidence promoting this device in PE; Difficulty in maneuvrability leading to incomplete thrombus removal; Large caliber increases the risk of RA perforation.	Difficulty in aspiration of large blood clots.

**Table 3:** Time-line of randomized controlled trials executed for catheter-directed therapies

	Randomized controlled trials
2014	ULTIMA: USCDL vs anticoagulation in intermediate-risk PE
2021	SUNSET sPE: USCDL vs CDL in intermediate-risk PE
2022	Kroupa et al CANARY trial: CDL vs anticoagulation in intermediate-risk PE
Future and Ongoing	PEERLESS: FlowTriever vs CDL in intermediate-risk PE PEERLESS II: FlowTriever vs anticoagulation in intermediate-risk PE STORM-PE: Indigo CAT16 vs anticoagulation STRATIFY: USCDL vs anticoagulation vs systemic thrombolysis in intermediate-risk PE HI-PEITHO: USCDL vs anticoagulation in intermediate-risk PE PE-TRACT: CDL or mechanical thrombectomy vs anticoagulation in intermediate-risk PE

The objective of the treatment in intermediate risk pulmonary embolism, is to prevent hemodynamic deterioration and instability, expedite the resolution of RV strain and prevent the progression to chronic thromboembolic pulmonary hypertension. We are considering patients that have a lower probability of mortality compared to high-risk patients, therefore the risk of bleeding that is associated with thrombolysis should also be weighed. For intermediate risk PE that is conventionally treated with anticoagulation, but often with poor clinical outcomes, the recruitment of aspirating devices could pave the way for better management of this grey-zone area. For the sake of this review, as in most studies, the efficacy of PE treatment will be evaluated based on clinical parameters, hemodynamics as well as right ventricular/left ventricular (RV/LV) ratio, and mean pulmonary artery systemic pressure (MAP) [11].

A previous meta-analysis with more that 2057 acute PE patients, concluded that systemic thrombolysis offered a lower mortality and lower recurrence rate benefit over anticoagulation only for high-risk patients but at a cost of higher rates of major hemorrhagic events including intracranial hemorrhage (ICH) [11]. Regarding intermediate risk patients, the pulmonary embolism thrombolysis trial (PEITHO) randomized more than 1000 patients to either thrombolysis or anticoagulation, concluding there was no difference in mortality among the two groups, although clinical deterioration was most frequent in the arm undergoing anticoagulation therapy and thrombolysis was associated with significant increase in major bleeding complications (8.7%) [12]. As such, much of the catheter directed lysis (CDL) treatment documentation stems from the successful clinical trials conducted on fibrinolytic systemic therapy, many of which are based on a small randomized clinical trial (RCT) and various short-term prospective single-arm studies [13].

The reason of administering lower doses of thrombolytics with CDL is to reduce the risk of intracranial bleeding, whilst improving the efficacy of the fibrinolytic drug by exposing a larger area of thrombus to degradation [13]. It was recently reported that systemic thrombolysis leads to a significantly higher rate of ICH and in-hospital mortality than CDL, with an overall estimated risk of ICH 1.5–3% [14, 15].

Catheter directed lysis enables the resolution of thromboembolic blockage in the pulmonary arteries, allowing restoration of pulmonary blood flow and leading to improved right ventricular function [9]. Unfortunately, due to the absence of high-quality evidence of available RCT’s examining the effectiveness of CDL and comparing its efficacy with the systemic fibrinolytic therapy in treating high-risk pulmonary embolism, CDL is currently not regarded as a first-line therapy for any risk category. However, in patients with high-risk PE that do not respond to systemic fibrinolysis, or when systemic thrombolysis is contraindicated, or for patients with clinical deterioration after anticoagulation, CDL should be suggested [9, 16].

This article aims to review the most recent up-to-date literature on the available CDT, including both CDL and mechanical thrombectomy devices, with a special focus on novel aspiration techniques.

### Catheter directed lysis

Catheter directed lysis (CDL) ranges from conventional CDL (cCDL) using only low-dose thrombolytic drugs, either tissue plasminogen activator (tPA) or Tenecteplase, and ultrasound assisted CDL (USCDL). In accordance with the clinical consensus based on the ESC guidelines for the percutaneous treatment of PE, only the catheter-directed ultrasound-assisted thrombolysis (USCDL) will be discussed as it is the newest treatment modality and is most frequently used within this category of devices and it has stronger evidence-based results. In contrast to systemic thrombolysis, where the drug is only delivered to the outer surfaces of the clot and often bypassed by collaterals developing around the occlusion, CDL ensures direct application of the lytic agent to the thrombus, with lower doses of tPA (approximately 0.01 mg/kg/h, typically ranging between 0.5 and 1 mg/h).

#### Technique

The only prospective randomized trial comparing USCDL with anticoagulation alone, the ultrasound-assisted catheter-directed thrombolysis (ULTIMA) RCT [17], utilized a standardized technique-wise approach that we will take as reference. EkoSonic MACH4e Endovascular Systems (EKOS Corporation, Bothell, WA) is the most studied device in the space and consists of the micro-ultrasound device and the intelligent drug delivery catheter, collected in a 6 F introducer for unilateral device or 10 F for a bilateral venous femoral approach [18]. It utilizes a dual lumen catheter allowing for the release of ultrasound energy locally from a core transducer, to enable the dissociation of fibrin strands and enhance the effects of local thrombolysis, separated by a marker from the infusion catheter portion. The infusion catheter is placed over a 0.035” guide-wire and accurately positioned at the treatment site. Once properly positioned, the guide-wire is replaced with the ultrasonic core into the catheter until the fittings firmly engage. The delivery of fibrinolysis and ultrasound halts at a mean duration of 15 h with a maximum administered dose of (tissue plasminogen activator) tPA at 20 ± 1 mg. Then, the ultrasonic core is taken out, and the guide-wire is reinserted into the catheter before removing everything.

#### Advantages and disadvantages

EKOS catheter-directed lysis therapy offers technical advantages such as precise clot targeting, enhanced thrombolysis with ultrasound, reduced systemic bleeding risk, minimized total thrombolytic dose, and improved recanalization rates. In addition, its small size reduces catheter-related complications at the access site and in the cardio-pulmonary circulation. In general, USCDL exhibits very low risk of bleeding in massive and sub-massive PE [19], with no major bleeding and three minor bleeding episodes [17], secondary to the lower total amount of thrombolytic needed and reduced systemic drug exposure, compared to systemic thrombolysis. However, the major limitations include longer procedure times and the need for closely monitored settings during infusion, such as an intensive care unit.

#### Evidence

ULTIMA, has shown that USCDL is superior to anticoagulation alone in the reversal of RV dilatation at 24 h without an increase in bleeding complications [17]. In addition, the CANARY trial comparing CDL to anticoagulation alone in intermediate-high risk patients [20], prematurely stopped due to the COVID-19 pandemic after enrolling 94 patients, found a significantly lower 72 h and 3-month RV/LV ratio with CDL than with anticoagulation (*p* = 0.01). The 3-month composite mortality was lower in patients treated with CDL.

Moreover, the randomized controlled SUNSET sPE trial [21], which compared USCDL with CDL in the management of intermediate PE, did not demonstrate any advantages over the latter. Instead, it slightly favoured CDL by reporting a significantly improved right ventricular/left ventricular ratio [21]. Moreover, two prospective clinical trials enrolling high-risk PE patients treated with CDL, such as the PERFECT evaluating the clinical success in terms of stabilization of hemodynamic status, improvement in pulmonary hypertension and/or right heart strain, and survival to hospital discharge [22], and SEATTLE II evaluating the RV/LV ratio modification at angio-CT scan, reported very promising results both in meeting the primary endpoints and the safety profile [19, 21]. However, the main limitations of ULTIMA, PERFECT, and SEATTLE II included the lack of a control arm (PERFECT and SEATTLE II), which does not allow the direct and reliable comparison of CDL to systemic thrombolysis or anticoagulation therapy, and the absence of clinical endpoints (SEATTLE II and ULTIMA) [23].

In the recent OPTALYSE PE trial evaluating the lowest possible dose of rt-Pa release from CDL in sub-massive PE to minimize the risk of intracranial hemorrhage, EkoSonic device was utilized for as little as 4 h, opening the opportunity for the faster mechanical thrombectomy procedure whilst maintaining efficiency and safety [24]. When comparing CDL with systemic thrombolysis in 339 patients with intermediate-risk PE in a retrospective study, authors observed lower mortality rates at 30 days (3% vs 10%) and at 1 year (8% vs 18%), without increase in bleeding rates [25]. The most recent research article retrospectively comparing EKOS with FlowTriever in 2259 patients [26], found a significant 28% lower rate of major bleeding events and 77% lower rate of intracerebral hemorrhage (0.3% incidence of ICH in patients treated with EKOS) in favour of EKOS. In addition, between the two devices there were comparable in-hospital mortality rates, 30-day all-cause readmission rates, and median lengths of hospital stay. However, authors stress the need for randomized trials to evaluate the safety and efficacy of CDL and comparing results with systemic thrombolysis, to determine the patients that would benefit most.

### Mechanical thrombectomy

Recently, new technologies have paved the way for large bore (8 F or more) endovascular aspiration catheters, able to rapidly remove proximal thrombi thus providing RV strain relief. The wide diameter of thrombectomy devices allow to effectively extract large organized and adherent emboli through aspiration. Clinical single-arm studies completed on these devices have shown a lower major bleeding incidence and mortality, significant reduction of RV dysfunction and PA pressure and lower mean stay in intensive care units, strengthening their role in determined clinical scenarios and creating opportunities for RCT’s [27–34]. This review will discuss the most studied products available.

#### The INARI Flow-Triever© system

The FlowTriever system (Inari Medical, Irvine, CA, United States) is an over-the-wire single use mechanical thrombectomy device that utilizes a large-bore (16 F, 20 F or 24 F) guide catheter and a nitinol expanding system that allows to engage the parietal thrombus facilitating suction.

##### Technique

**Fig. 1:**
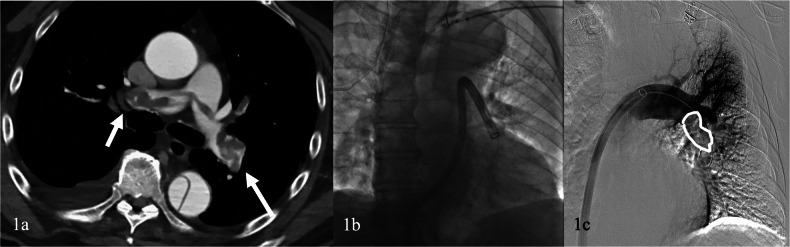
A 68-year-old patient presented to the emergency department hypotensive with saturation drop. **a** Computed tomography angiography confirmed the presence of bilateral extensive pulmonary emboli (arrows). **b** Treatment with INARI 20 F device followed with numerous bilateral aspirations. **c** Contrast injection confirmed the reduction of clot burden (white draw line). The nitinol expanding system was not required

**Fig. 2:**
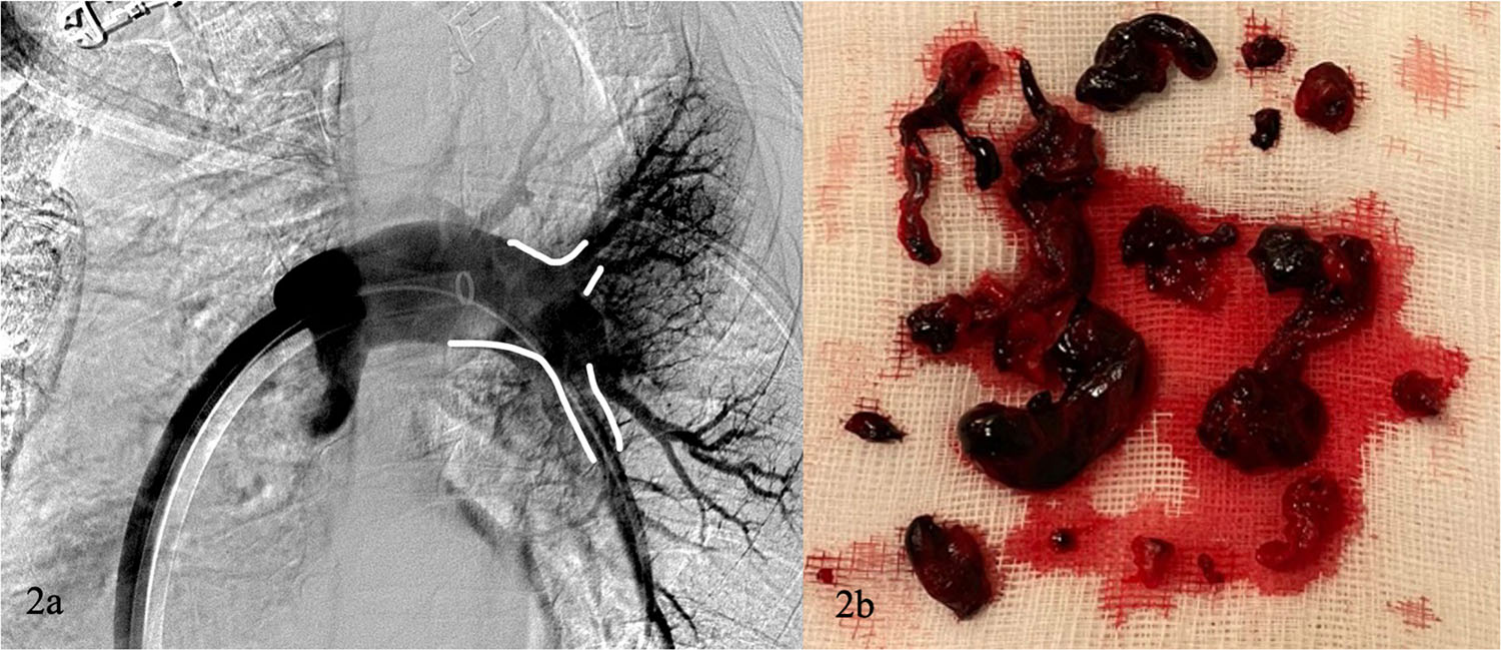
Previous patient. **a** Final diagnostic angiographic control shows complete patency of the pulmonary arteries (white draw line). Significant reduction of pulmonary artery pressure and RV/LV ratio at 48 h was observed. **b** Chronic and acute clot elements were extracted

Initial access is through puncture of the common femoral vein, followed by insertion of a 7-F sheath, using standard technique. A 0.035-inch guidewire is advanced distally into a lobar or segmental pulmonary artery and then exchanged for a stiff guide-wire. A large 20/22-F introducer sheath is swapped and advanced to the suprarenal inferior vena cava (IVC). The 20-F aspiration guide catheter (AGC) is advanced over the wire into a main or lobar pulmonary artery just proximal to the occlusive thrombus. Aspiration of the thrombus is performed by applying negative suction to the AGC through a 60-cm^3^ locking syringe (Figs. 1, 2). Should the aspiration be unsuccessful with the AGC alone, the three self-expanding nitinol discs of the FlowTriever catheter can be deployed through the AGC into the targeted thrombus in order to facilitate the clot engagement. Aspiration thrombectomy should be repeated until reaching the correct clot removal.

##### Advantages and disadvantages

The strong points of INARI FlowTriever are its design, which facilitates the trackability through the right atrium and pulmonary trunk, with a shaft able to withstand kink resistance and a caliber large enough to efficiently suction the thromboembolic material. The main difficulties of this device include aspiration-related blood loss (which decreases with operators’ experience) and damage to cardiac structures or small distal pulmonary arteries, partly due to the large size and stiffness of the catheter. In this regard, recently INARI released the Triever16 Curve that offers advantages over continuous aspiration devices, including a pre-built curve for target aspiration and compatibility with the FlowSaver blood return system.

##### Evidence

The prospective FLARE multicenter trial evaluating 107 patients with intermediate-risk PE, reported a significant RV/LV ratio improvement, despite 6 major adverse events (MAEs), including 1 death (1%) at 30-day follow-up however due to unrelated reasons [35]. Furthermore, recent interim-results on 57 patients based on the analysis of the prospective multicenter FLASH registry aimed to analyze the safety profile and efficacy in 800 patients, evaluating real-world outcomes in patients predominantly intermediate-risk PE, reported no device-related mortality, a 0% mortality rate at 48 h at the cost of 1.2% rate of bleeding event, and 0.4% of all-cause mortality at 30 days [27]. Significant reduction in both MAP (18.8%) peri-procedurally and the RV-LV ratio at 48 h (29.9%) was observed, among other clinical symptoms such as dyspnea that significantly improved throughout the follow-up periods.

Moreover, FLAME is the largest prospective study evaluating the efficacy of FlowTriever, compared to the context arm with other contemporary therapies (68.9% systemic thrombolytics), in 115 high-risk PE [28]. Precisely, it demonstrated a 90% survival improvement, precisely in-hospital mortality was 1.9% in the FlowTriever arm compared to 29.5% in the context arm for other therapies [28]. In addition, thrombectomy was associated with significantly lower occurrence of in-hospital meaningful MAEs including ICH.

By enrolling over 1700 patients, PEERLESS and PEERLESS II studies, are conducting the largest randomized clinical trials investigating the treatment in intermediate-high-risk PE. PEERLESS and PEERLESS II, aim to compare FlowTriever to CDL in an estimated 550 patients and FlowTriever with anticoagulation in 1200 patients, respectively. It is the first clinical trial that compares interventional therapies in acute PE. Primary end-points focus on clinical mortality, MAEs and clinical deterioration among others [36].

#### The AngioVac© system

The utilization of the AngioVac (Angiodynamics, USA) suction thrombectomy system, for PE treatment has been constrained, due to the predominant district application indication, but not limited to, including iliofemoral vein, IVC, superior vena cava and right heart, the substantial allocation of resources that it demands, and the few positive results published in the treatment of this disease. It was constructed and approved by FDA to remove venous thrombi or emboli during extracorporeal venous-venous bypass. Commonly it is also used for ilio-caval thrombi in 53%, right side heart thrombus in 49%, and occasionally off-label in PE [37, 38].

##### Technique

The Angiovac system consists of an 18 F or 22 F coil reinforced cannula with a balloon actuated expandable funnel-shaped distal tip. The catheter is part of a venous-venous recirculation extracorporeal system allowing removal of the aspirated thrombus while returning blood to the patient to maintain hemodynamic stability. Either femoral or jugular vein is punctured and after several sheath dilations, a 26 F GORE sheath is introduced and a super stiff guide-wire is navigated to the region of interest upon which the catheter is placed, under fluoroscopic guidance or echocardiography in case of atrial thrombus. The catheter is then temporarily clamped and an additional venous access is obtained to make home for a 17 F or a 19 F reperfusion catheter, after to which a filter is applied to complete the circuit. Aspiration is initiated once the aspirating cannula is placed on the face of the thrombus, requiring also a continuous advancement and withdrawal to facilitate the aspiration. Extracorporeal venous-venous blood circulation is sustained for up to 6 h by blood suction from the AngioVac catheter generating flow rates up to 4 liters per minute, after which it is returned to the patient by the reperfusion catheter.

##### Advantages and disadvantages

Due to the current design of the AngioVac system, related to an inadequate stiffness and caliber that increase the difficulty of maneuver and steerability, several studies reported incomplete evacuation of the venous emboli in PE, with success rates between 12.5 and 33% [29, 37, 39]. Navigation via the right atrium is not easy with this system and there is a significant risk of perforation, therefore advancement of the cannula requires the support of an extra stiff guidewire. Other difficulties were encountered in the case of complete blockage due to thrombus, where flow rates might be insufficient for effective extraction. Advantages include the ability of this system is to remove clots from other large venous districts such as in an IVC filter, or in case of pacemaker or valvular bound thrombi [40, 41]. Also, due to the venous-venous extracorporeal system only few bleeding events occurred [29]. Concomitant deep vein thrombosis (roughly in 50% of proximal PE cases) is associated with 4-times increase of both mortality at 90 days, thus the ability of this device to act also in the deep venous system could theoretically improve mortality in this population [42].

##### Evidence

Few reports have been published on the use of AngioVac device in PE, with little specific outcomes retrievable [43]. There is a lack of evidence in promoting the use of this device; no clinical trials nor strong large observational studies have been released. Published mortality rates at 30-days were 13%, however the population included also right heart thrombi which are associated with a higher mortality rate compared to PE in case of treatment with anticoagulation [29]. A single center study on 5 patients with intermediate-high-risk PE reported an overall mortality rate in the follow-up period of 80%, including one case secondary to RV wall perforation [30].

#### The INDIGO© system

The Penumbra system (Penumbra Inc., CA, United States) is a suction aspiration system first used in the endovascular treatment of stroke. The latest commercially available Penumbra Indigo aspiration systems are the lightning intelligent aspiration catheter (CAT) 8, CAT12 and CAT16, the latter just approved by the FDA. The aspiration system received approval in the treatment of peripheral arterial system and PE by the FDA in 2021.

##### Technique

**Fig. 3:**
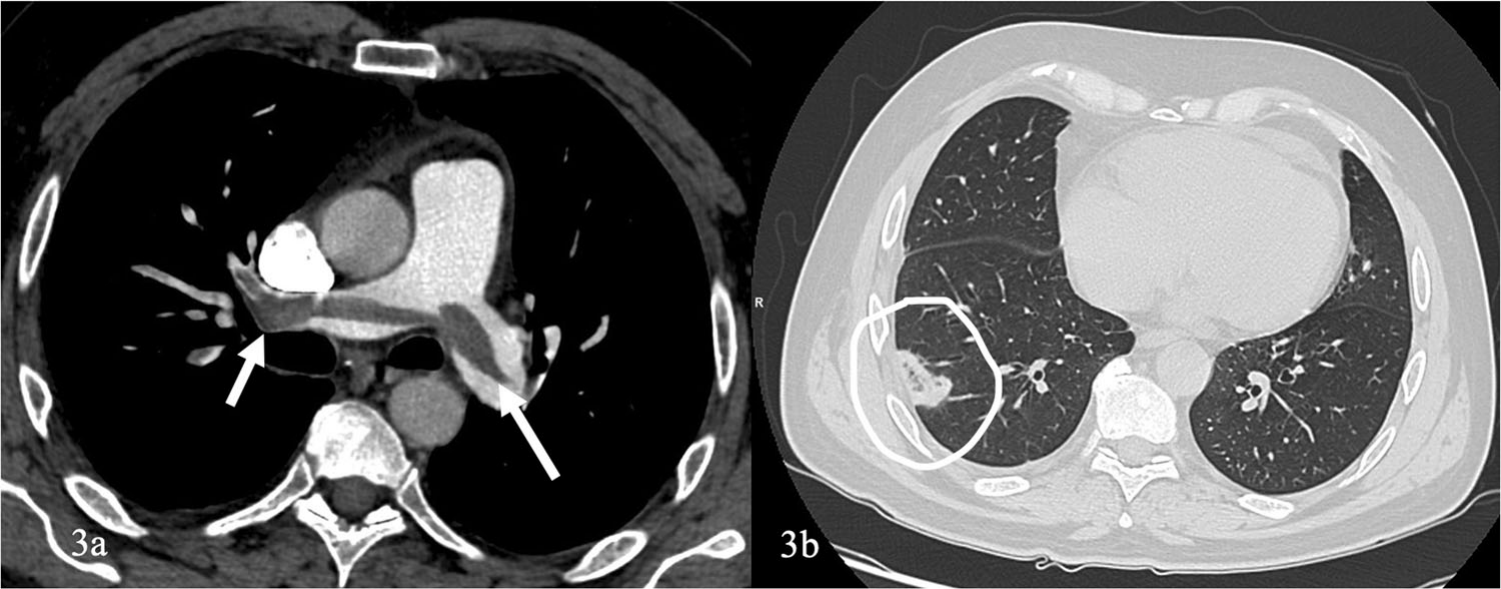
**a** Computed tomography angiogram scan of a 73-year-old that presented to the emergency department with deep vein thrombosis, hypotensive and with a drop of oxygen saturation, that confirms extensive bilateral pulmonary thrombus (arrows). **b** Pulmonary infarct is revealed in the right lower lobe (white circle)

**Fig. 4:**
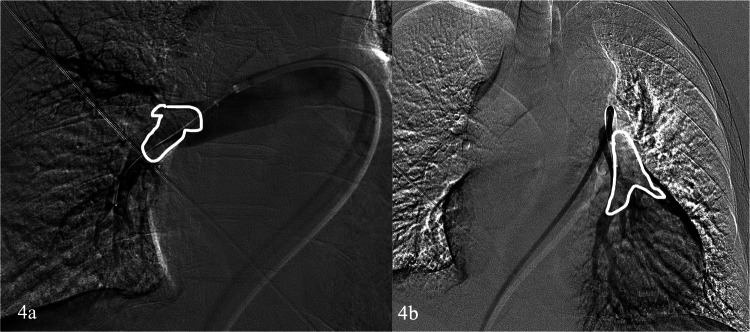
The Fig. 3 patient was treated with the INDIGO Penumbra Lightning CAT8 device. **a**, **b** angiographic evaluation confirms filling defects (white draw line) that correspond to the thrombus and numerous aspirations followed at that level bilaterally

**Fig. 5:**
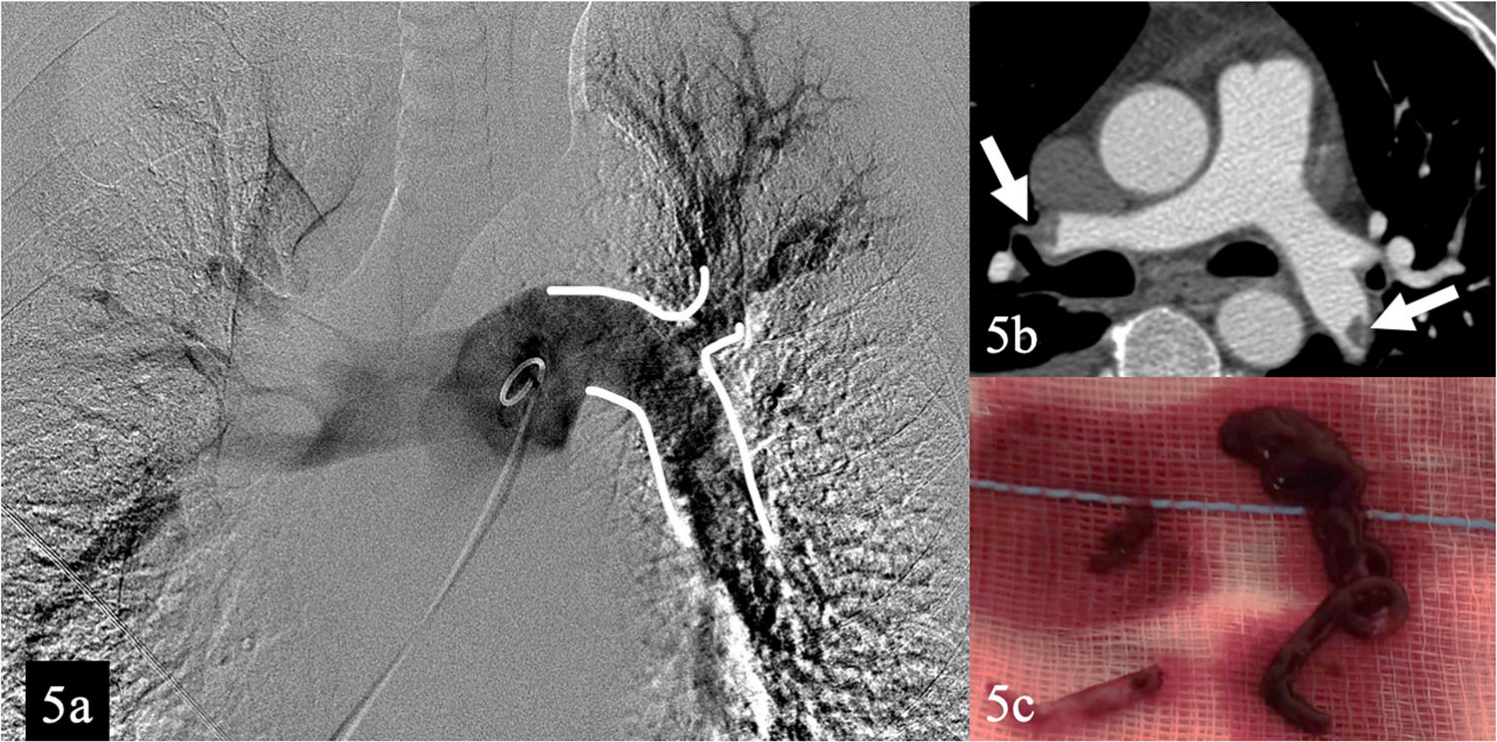
Previous patient (**a**) Pigtail angiogram confirms satisfactory clot extraction with patency of the pulmonary arteries (white draw line). Significant reduction of pulmonary artery pressure and RV/LV was observed at 48 h. **b** Computed tomography angiogram confirms only small volume residual thrombus (arrows). **c** Chronic and acute clot elements were extracted

Dual femoral venous puncture access with 6–8 F introducer sheath is usually performed. Normally, a 6 F Pigtail catheter is advanced on one end towards the pulmonary trunk to perform an angiography, allowing to characterize with more detail the presence and extension of the embolus. The standard 0.035-inch guidewire is exchanged to an 0.035 inch super-stiff and a long introducer sheath of at least 12 F is positioned proximally to the pulmonary trunk. Then, the aspiration catheter is advanced to the thrombus and aspiration is initiated. During the aspiration, if prolonged contact with the embolus is requested, the guidewire can be removed (Figs. 3, 4, and 5).

##### Advantages and disadvantages

With respect to the obsolete Indigo CAT8, its newer version Lightning, made of steel with a robust hypotube technology and laser cut design, is able to develop high power and flexibility. This state-of-the-art design favours trackability and torqueability from tip to base, improving maneuverability in tortuous vessels and in large ventricles. In addition, advancing technology of the lightning system in CAT8 and CAT12 processors, enabled the control of blood loss during the aspiration process, thanks to high-frequency valves and a system capable of measuring the pressure and flow of the fluid. This pulsing technique significantly reduces blood loss, estimated at 18:1 reduction in fluid loss, compared to the original Dynamic Aspiration Tubing [31].

A possible disadvantage of the relatively smaller size of penumbra catheters, compared to other aspiration devices, is the difficulty in aspirating larger clots. Nevertheless, the smaller size reduces the risk of vascular access complications or damage to the pulmonary artery or right atrium throughout the procedure.

#### Evidence

A Prospective, Multicenter Trial to Evaluate the Safety and Efficacy of the CAT 8 Indigo Aspiration System in Acute Pulmonary Embolism (EXTRACT-PE) in 122 patients, demonstrated a significant reduction (mean 28%) of the RV/LV ratio at 48 h (0.43) and a significant reduction of intraprocedural PA pressure [44]. The mean time of utilization of the indigo device system was the shortest yet to be reported (37 min). This system proved its efficacy not only in the main pulmonary artery but also distally in pulmonary branches [32]. Finally, and most importantly, only three MAEs including hemoptysis were registered, one death occurring secondary to access site complications. No intracranial bleeding events were reported [44]. As a result of the EXTRACT-PE, in USA, CAT8 was elected for the treatment of PE thereby promoting research developments for more efficient catheters, these being the intelligent lightning aspiration system CAT8 and CAT12. In addition, a most recent CATH-PE investigation on 100 intermediate-high-risk and high-risk PE treated with CAT8 system bilaterally in pulmonary arteries, obtained similar results, including a significant reduction of pulmonary artery pressure (PAP) and RV/LV ratio at 24–48 h [34].

Later, the ongoing STRIKE-PE single-arm prospective trial [34], utilizing Lightning12, was launched to evaluate the reproducibility of the EXTRACT trial in 55 different locations both in US and EU for a total of 600 patients, in addition to assessing various clinical parameters. The most recent analysis of interim results by Perkowski et al on 60 patients (95% intermediate-risk PE; 5% high-risk PE) [45], have elucidated the advantages regarding the additional dual mechanism of the aspiration system, now based on pressure and flow. Both RV/LV ratio at 48 h and peri-procedural systolic PAP significantly decreased, of 24.3% and 19.9%, respectively. Furthermore, the short thrombectomy time (36 min), in conjunction with the absence of death or symptomatic PE recurrence at 30 days and the improvement of Borg dyspnoea score at rest at 90 days, outweighed MAEs such as access site hematomas even though in some cases blood transfusions were required. Last, future RCTs are awaiting initiation, the first being STORM-PE comparing Flash lightning CAT16 with anticoagulation in 100 patients; the primary endpoint being RV-LV ratio at 48 h [46]. Second, another RCT is awaiting initiation comparing CAT8 to hydro-mechanical defragmentation utilizing pig-tail catheter in 200 intermediate-high risk patients [47].

### Future perspectives

Mechanical thrombectomy for PE is undergoing rapid advancements, both in the novelties brought by the manufacturers and in clinical evidence to guide treatment choices. As such, taking into account the important results obtained in ongoing randomized trials, thrombectomy is gaining authority and popularity in the treatment of specific clinical conditions. However, the main issue is to tailor treatment for patients given the variety of available options and clinical scenarios. While randomized trials are essential for evidence-based decision making, it’s impractical to compare each method of mechanical thrombectomy with each other or with systemic therapy for every PE scenario. Although RCT’s comparing aspiration catheters to other therapies are ongoing, to the best of our knowledge to date there are no completed RCT’s comparing mechanical thrombo-aspiration devices to other therapies, leaving clinicians to base their decisions on well-conducted prospective single-arm studies.

While acute PE studies highlighting clinical improvements often focus on short-term outcomes, the long-term effects and the association with chronic thromboembolic pulmonary hypertension and functional impairment in survivors are crucial but challenging to investigate. In conclusion, aspirational thrombectomy for PE may offer a valid solution for both patients with intermediate and high risk by preventing immediate hemodynamic deterioration and by reducing post-hospital mortality rates. The use of thrombolysis is less frequently necessary as the clot may be quickly removed mechanically in most of the cases. The field is evolving rapidly with ongoing trials, but the main challenge is integrating this growing evidence into individualized care pathways for the diverse needs of acute PE patients.

### Objective

This review discusses the most recent data on the endovascular methods currently available for the treatment of intermediate and high-risk pulmonary embolism (PE), with a special focus on mechanical thrombectomy. Systemic thrombolysis does not appear sufficient to reduce mortality in patients who develop hemodynamic instability and is inevitably linked with significant side-effects such as intracranial hemorrhage (ICH) and other major adverse effects (MAEs). Furthermore, even patients who initially appear stable may deteriorate and become unstable if PE is extensive. Aspiration thrombectomy may offer a valid solution for such patients offering fast clinical improvement without the high risk of hemorrhagic complications, as demonstrated by several single-arm studies and interim-results of randomized controlled trials (RCTs). We haven’t yet reached a post-fibrinolytic era however there is no doubt that there is a number of valid solutions available that need to be taken into consideration for the management of such patients.

## Conclusions

Efficacy and safety of endovascular aspiration devices in intermediate- and high-risk PE have been demonstrated. Reproducibility of these case-series studies and randomized-control trials comparing the use of these devices with other available methods are necessary in this rapidly evolving era of aspirating catheters; ongoing and promising interim-results have been published. Should the beneficial properties of aspiration techniques be confirmed, future randomized trials comparing them with one another will be necessary.

## Abbreviations

CAT: Aspiration catheter
CDL: Catheter directed lysis
CDT: Catheter directed therapy
ESC: European Society of Cardiology
ICH: Intracranial hemorrhage
MAEs: Major adverse events
MAP: Mean pulmonary artery systemic pressure
PAP: Pulmonary artery pressure
PE: Pulmonary embolism
RCT: Randomized clinical trial
RV: Right ventricle
RV/LV ratio: Right ventricular/left ventricular
USCDL: Catheter-directed ultrasound-assisted thrombolysis
